# Upper Limb Sensory-Motor Control During Exposure to Different Mechanical Environments in Multiple Sclerosis Subjects With No Clinical Disability

**DOI:** 10.3389/fnbot.2022.920118

**Published:** 2022-07-11

**Authors:** Camilla Pierella, Laura Pellegrino, Margit Muller, Matilde Inglese, Claudio Solaro, Martina Coscia, Maura Casadio

**Affiliations:** ^1^Department of Neurosciences, Rehabilitation, Ophthalmology, Genetics, and Maternal and Children's Sciences (DINOGMI), University of Genoa, Genoa, Italy; ^2^Department of Informatics, Bioengineering, Robotics and Systems Engineering (DIBRIS), University of Genoa, Genoa, Italy; ^3^Department of Rehabilitation, C.R.R.F. “Mons. L. Novarese”, Moncrivello, Italy; ^4^IRCCS Ospedale Policlinico San Martino, Genoa, Italy; ^5^confinis ag, Düdingen, Switzerland

**Keywords:** motor control, multiple sclerosis, upper limb, robotic assessment, muscle synergies, spinal maps

## Abstract

Multiple sclerosis (MS) is an autoimmune and neurodegenerative disease resulting in motor impairments associated with muscle weakness and lack of movement coordination. The goal of this work was to quantify upper limb motor deficits in asymptomatic MS subjects with a robot-based assessment including performance and muscle synergies analysis. A total of 7 subjects (MS: 3 M−4 F; 42 ± 10 years) with clinically definite MS according to McDonald criteria, but with no clinical disability, and 7 age- and sex-matched subjects without a history of neurological disorders participated in the study. All subjects controlled a cursor on the computer screen by moving their hand or applying forces in 8 coplanar directions at their self-selected speed. They grasped the handle of a robotic planar manipulandum that generated four different environments: null, assistive or resistive forces, and rigid constraint. Simultaneously, the activity of 15 upper body muscles was recorded. Asymptomatic MS subjects generated less smooth and less accurate cursor trajectories than control subjects in controlling a force profile, while the end-point error was significantly different also in the other environments. The EMG analysis revealed different muscle activation patterns in MS subjects when exerting isometric forces or when moving in presence of external forces generated by a robot. While the two populations had the same number and similar structure of muscle synergies, they had different activation profiles. These results suggested that a task requiring to control forces against a rigid environment allows better than movement tasks to detect early sensory-motor signs related to the onset of symptoms of multiple sclerosis and to differentiate between stages of the disease.

## Introduction

Multiple sclerosis (MS) is the most widespread disabling neurological condition of young adults around the world (Browne et al., 2014; Thompson et al., 2018). Nearly 75% of people with MS experience upper limb dysfunctions such as tremor, coordination deficits, and muscle weakness (Bertoni et al., 2015; Thompson et al., 2018; Valè et al., 2020). These symptoms severely reduce their quality of life (Mokkink et al., 2015; Thompson et al., 2018), which might be further impaired by the rise of pain and fatigue worsening with the progression of the disease over time (Kister et al., 2013). Because of the high risk of future disability, the detection of the onset of subtle functional impairments is crucial in the early stages and in the asymptomatic phase of the disease. Asymptomatic MS describes a clinically silent disease state of MS usually incidentally discovered after imaging or other diagnostic exams performed, for example, for routine check-ups. Magnetic resonance imaging (MRI) and clinical tests have suggested that in asymptomatic MS subjects despite the morphological evidence of subclinical disease, the CNS damage remains very limited, and unknown protective mechanisms prevent its clinical manifestation in these individuals (Stefano et al., 2006; Siva, 2013; Amato et al., 2022).

In clinical practice, standard tests like Expanded Disability Status Scale (EDSS) (Yozbatiran et al., 2006) are used to evaluate motor impairments. Standard tests provide a global assessment and not a fine characterization of the movement. This is a limitation for a disease such as MS that can present a wide range of symptoms. Given the variety of disabilities in MS, outcome measures should be able to capture multiple clinical dimensions (Uitdehaag, 2014). Technology-based, i.e., robotic-based assessment can help, not only to train but also to well characterize and quantify motor impairment following a neurological injury or disease (Padua et al., 2007; Reinkensmeyer and Boninger, 2012; Lamers et al., 2016). In the last decades, robots have been proven to successfully help physiotherapists in MS rehabilitation (Feys et al., 2015; Lamers et al., 2016; Boffa et al., 2019; Gandolfi et al., 2019), given the advantages of high-intensity training, for volume and duration, and higher controllability of the training environment. And also in other pathologies, robotic rehabilitation played a crucial role in reducing upper limb dysfunctions, improving manual dexterity, arm strength, and performance of the activities of daily living (ADL) (Lamers et al., 2016; Halabchi et al., 2017; Kubsik-Gidlewska et al., 2017). Despite their potential, robots have been mostly used for rehabilitation and not as a tool to support and develop assessment protocols able to increase the knowledge of the mechanisms underlying the impairment of upper body functions after MS. Robots are excellent platforms to control the task, providing different external forces and allowing quantitative and repeatable motor performance measures that can be used to assess motor recovery in people with MS (Casadio et al., 2007; Solaro et al., 2007; Carpinella et al., 2014; Pellegrino et al., 2015, 2018; Simmatis et al., 2020; Valè et al., 2020). Robotic set-ups have been used with people with MS while performing planar movement in different mechanical environments using an end-effector robot (Padua et al., 2007; Solaro et al., 2007; Vergaro et al., 2010; Carpinella et al., 2012; Pellegrino et al., 2018), or while MS subjects were performing tasks in 3D, instrumenting upper limb with EMG sensors and/or motion capture trackers (Padua et al., 2007; Pellegrino et al., 2015; Lamers et al., 2016; Gandolfi et al., 2019; Valè et al., 2020). A multimodal assessment of kinematic and muscle activations allows to consider motor control and behavior, particularly relevant for neurological diseases as neural deficits may be masked at the kinematic level by compensatory strategies and kinematic impairments are a result of the effect of the neural deficits on muscle activation. The evaluation of behavioral parameters together with the measure of neurophysiological signals, such as the muscular (EMG) activity, and the extraction of muscle synergies opened the possibility for a comprehensive characterization of the origin, the expected prognosis, and the functional consequences of motor impairments after symptomatic MS. Muscle synergies, by describing how groups of muscles activate together while performing a motor task, have been proven to be a good descriptor of motor coordination in unimpaired individuals and in people with neurological diseases (Cheung et al., 2009, 2012; Tropea et al., 2013; Ting et al., 2015; Torricelli et al., 2016; Pellegrino et al., 2018, 2021a). At the same time, they were able to differentiate between normal and pathological behavior in different conditions like stroke (Cheung et al., 2009; Tropea et al., 2013; Pellegrino et al., 2021b), MS (Lencioni et al., 2016; Pellegrino et al., 2018), or spinal cord injury (Barroso et al., 2015), resulting in a useful clinical tool (Safavynia, 2011; Torricelli et al., 2016).

We hypothesize that an instrumented, controlled, and multimodal assessment based on a robot-assisted analysis supported by EMG data collection and analysis, such as muscle synergies analysis, is able to detect minimal subtle abnormalities in upper limb movements. We expect this to be particularly relevant when MS is asymptomatic and the standard clinical tests like EDSS are not able to detect motor impairment, and only magnetic resonance imaging is detecting abnormalities in the white matter of such asymptomatic subjects (Pontillo et al., 2022; Schiavi et al., 2022; Zhang et al., 2022). This method can provide objective data, which could be then utilized for the quantitative evaluation of the course of the disease or of the response to specific therapeutic strategies. So far, this approach has not been fully exploited to study asymptomatic MS (Solaro et al., 2007; Casadio et al., 2008).

Therefore, the goal of this study was to identify and characterize deficits in motor control and behavior of the asymptomatic MS population in different dynamic and isometric planar tasks. Starting from a method grounded in the literature and already tested with symptomatic MS and stroke subjects (Pellegrino et al., 2018, 2020, 2021b) based on a robotic manipulandum to control reaching movements for a quantitative and repeatable assessment of upper limb kinematics, we investigated how kinematics and muscle activity were affected by the disease and by task features, in particular when interaction forces change.

## Materials and Methods

### Participants

A total of seven subjects with clinically definite MS according to McDonald (MS: 3 M−4 F; 42 ± 10 years; 3 subjects with EDSS = 1 and 4 subjects with EDSS = 0) with no clinical disability, and seven healthy age- and sex-matched control subjects (C: 3 M−4 F; 42 ± 9 years) participated in this study. All subjects were right-handed, i.e., for all subjects, the right and left sides of the body were respectively the dominant (D) and non-dominant side (ND). Control subjects did not present any evidence or known history of skeletal or neurological diseases, and they exhibited intact joint range of motion and muscle strength. Inclusion criteria for MS subjects were the following: clinically definite MS according to McDonald criteria; Expanded Disability Status Scale (EDSS < = 1); Fatigue Severity Scale (FSS ≤ 20); presence of neurological signs only, but no signs or symptoms at upper limbs and consequently, “normal” score for the “arm” portion of the Scripps' neurological rating scale (Koziol et al., 1999) for the sensory (3 out of 3), motor (5 out of 5), and cerebellar (5 out of 5) systems; stable phase of the disease (no relapses in the last 3 months). The exclusion criteria were: mini-mental state examination (MMSE) <28, treatment with corticosteroids within the previous 3 months, and symptomatic oculomotor signs or visual acuity <8/10.

The study was approved by the local Ethical Committee (Comitato Etico Regionale Liguria, 06-10-2014, 201REG2014) and conformed to the ethical standards of the 2013 Declaration of Helsinki. Each subject provided written informed consent to participate in the study and to publish individual data.

### Experimental Set-Up and Procedure

The protocol consisted of a single session of tests lasting about 2 h. Subjects sat on a chair and grasped the handle of the planar robotic manipulandum (Casadio et al., 2006), Figure 1A. The robot had low friction and inertia, zero backslash, and was actuated by a pair of direct-drive brushless electric motors.

**Figure 1:**
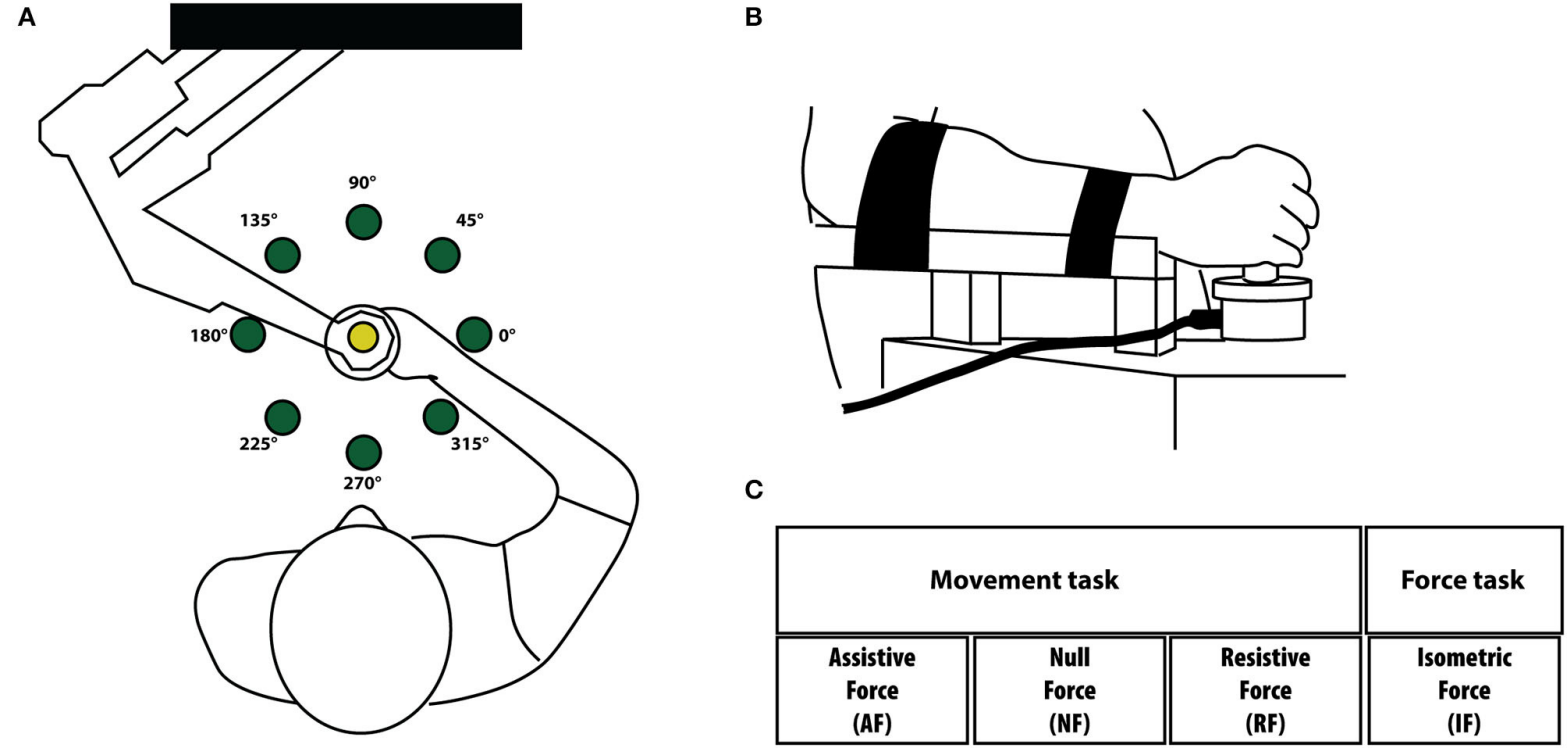
**(A)** Experimental setup for the movement tasks. Subjects held the handle of the planar robotic manipulandum and made reaching movements toward eight targets presented on a computer screen placed in front of them. The targets are shown as green circles. **(B)** In the force task, subjects did not move their arm and hold with their hand a fix force sensor. The movement of the cursor in the screen was controlled by the isometric force applied by the subjects to the force sensor. Targets and directions were the same in both tasks. We considered equivalent directions that corresponded in joint coordinates and the left-arm movement/forces (and corresponding cursor trajectories) were mirrored at the midline in the endpoint space. **(C)** Experimental protocol. Tasks were performed in four different mechanical environments: assistive force (AF), null force (NF), resistive force (RF) and isometric force (IF). Subjects executed the task with their right-dominant (D) and left-non- dominant (ND) arms.

Their forearm and wrist were restrained by means of suitable holders to remove the effect of the gravity and to limit prono-supination strategies. The position of the seat was adjusted in such a way that the movements were restricted to the horizontal plane. A chair provided secure back support and two belts prevented appreciable trunk movements. A 19-inch LCD computer screen was placed vertically in front of the subjects, around one meter away, at eye level.

MS and control subjects had to control a cursor moving on the screen. In the dynamic tasks, the cursor motion was controlled by the movement of the hand grasping the manipulandum. In the isometric case (Figure 1B), the cursor was controlled by the two components of the force in the same plane (i.e., the plane parallel to the floor). About 1 N of force exerted on the sensor caused a shift of 1.4 cm of the cursor position on the screen. The robot encoders recorded the end-effector position. A force sensor (Gamma SI 13010, ATI Industrial Automation Inc.) restrained to a fixed position measured the force applied to it in the isometric tasks.

Participants performed the experiment with their right-dominant (D) and left-non dominant (ND) hands separately. Subjects interacted with four different mechanical environments as in (Pellegrino et al., 2018, 2021b), Figure 1C. In particular:

1- Assistive force field (AF). A constant assistive force field attracted the hand of the subjects toward the peripheral target (force amplitude: 5 N).
2- Null force field (NF). The hand of the subjects moved in the workspace with no external forces acting on it.
3- Resistive force field (RF). A resistive force field attracted the hand of the subjects toward the center of the workspace, i.e., an elastic force opposed the subjects' movements toward the peripheral targets (linear spring stiffness coefficient was 15 N/m).
4- Isometric force task (IF). The subject's hand applied isometric forces to reach the peripheral targets. To note that in this task subjects held a fixed force sensor instead of the handle of the manipulandum.

The task consisted in reaching eight targets positioned in eight equally spaced directions and placed 14 cm away from a central target. This distance corresponded to a displacement of 14 cm of the end-effector in the FS, AF, and RF tasks, and to a 10 N force step for the IF task; Figure 1B. Each target (green circle, 10 mm radius) was presented five times in random order. Therefore, subjects performed 40 center-out movements per task, for a total of 160 center-out movements for each arm. Each target was presented again only after all eight targets had been reached. The cursor (yellow circle, 5 mm radius) position corresponding to the motion or the force applied at the robot end-effector was continuously displayed during the execution of all tasks. All subjects started the experiment with the dominant (right) arm. The different tasks were presented in random order within each arm. Subjects were asked to reach the targets as accurately as possible, at their self-selected speed (no time constrain). The protocol required a minimum of 2 min break between each task. Subjects were allowed to rest when and as long as they needed.

We recorded muscle activity of 15 muscles from each upper limb with surface electrodes for electromyography (CometaWavePlus, Cometa Srl, Italy). Electrodes were placed according to the guidelines of the Surface Electromyography for the Non-Invasive Assessment of Muscles European Community project–SENIAM (Hermens et al., 2000) and Anatomical guideline (Perotto and Delagi, 2005) on the following muscles like in previous studies (Pellegrino et al., 2018, 2021a,b): triceps brachii long (TB-long) and lateral head (TB-lat), biceps brachii short (BB-short) and long head (BB-long), brachioradialis (BRAD), brachialis (BRA), pronator teres (PRON), infraspinatus (INFR), latissimus dorsi (LAT), upper trapezius (TRAP), rhomboid major (RHOM), pectoralis major (PECT), anterior (DELT-ant), medial (DELT-mid) and posterior (DELT-post) deltoid.

### Data Analysis

#### Task Performance

Movement and force trajectories were sampled at 60 Hz and smoothed by using a 6th order Savitzky–Golay filter (cutoff frequency: 11 Hz for the movement signals and 8 Hz for the force signals), which was also used to estimate the subsequent time derivatives of the trajectory. A lower cutoff frequency was chosen for the force because the signals read from the force sensor were noisier than the movement signals read from the encoders. We focused on the center-out cursor movements, i.e., each trial considered in the movements from the moment in which the cursor is in the central target until the moment in which the cursor is in the peripheral target. The cursor movement onset was defined as the first time instant in which the cursor speed exceeded the threshold of the 10% of the maximum peak speed for the movement task while (Casadio et al., 2008) the movement ended when the cursor was inside the target and its speed underwent and remained under the same threshold (Casadio et al., 2008). We analyzed the following performance indicators:

- Average speed (m/s)–average speed of the cursor movement.
- Normalized Jerk index (adimensional)–the square root of the jerk; i.e., the third time derivative of the cursor position, averaged over the entire movement duration and normalized with respect to cursor movement duration (MD) and path length (PL) (Teulings et al., 1997).

$$\begin{array}{l} Normalized\;Jerk=\sqrt{\frac{1}{2}\int dtj^{2}*\frac{MD^{5}}{PL^{2}}} \end{array}$$

This indicator is a measure of smoothness, high values correspond to jerky movements, indicating impaired motor control.

- 100-ms aiming error (deg)–the angular difference between the target direction and the actual movement direction, estimated in the first 100 ms of the movement or of the force exertion (Casadio et al., 2007, 2008).

It indicates the ability to plan the movement, i.e., feed-forward component of motor control.

- End-point error (m)–the distance between target and cursor position when the speed of the cursor, after reaching a peak, felt for the first time below 10% of the maximum speed of that movement trial (Casadio et al., 2007). It indicates accuracy in the execution of the movement.

#### EMG Analysis

##### Preprocessing and Muscle Activations

EMG signals were acquired at 2 kHz, band-pass-filtered (30–550 Hz), rectified, and then low-pass-filtered (cutoff: 10 Hz) to obtain the EMG envelopes (Cheung et al., 2009, 2012). The envelope of each muscle signal was normalized by its median value obtained during the center-out cursor movements of all the tasks, i.e., for each subject, we computed the median over all his/her EMG signals considered in the study. The normalization based on the median value instead of the maximum is more robust against high-amplitude spikes arising from noise (Cheung et al., 2009). The normalized EMG envelopes for each subject, arm, task, and repetition were segmented according to the 8 task directions.

##### Spinal Maps

Pre-processed EMG signals were used to estimate the correspondent spatiotemporal organization of the MN activity in the spinal cord (Yakovenko et al., 2002; Coscia et al., 2015; Pirondini et al., 2016; Pierella et al., 2020). To characterize the spinal motor output, EMG activity was mapped onto the estimated location of MN pools innervating the different muscles of the upper limb using weighting coefficients as reported by Kendall (McCreary and Provance, 1993), Supplementary Table 1. Specifically, for each spinal segment, the indirect measure of MN activity was computed as the weighted summation of all EMG signals innervated by such segment, where weight coefficients were those tabled in literature (McCreary and Provance, 1993) and reported in Supplementary Table 1. The map was limited to levels between C2 and T1 in relation to the set of recorded muscles. To compute the spinal maps, the normalized EMG envelope of each muscle related to each trial was resampled on 100 time points. To compute the spinal map, S, for each segment, j, we used the following equation: $S_{j}=\frac{\overset{n_{j}}{\underset{i=1}{\sum }}k_{i,j}EMG_{i}}{n_{j}}$; where n_j_ is the number of EMGi waveforms corresponding to the jth segment and k_i,j_ is the weighting coefficient of the ith muscles following the values reported in the Kendall's chart. To describe the similarity between two spinal maps, we used the 2D Pearson's correlation coefficient (ρ_2D−GROUP_) (Yakovenko et al., 2002; Coscia et al., 2015; Pirondini et al., 2016; Pierella et al., 2020). The averaged 2D Pearson's correlation coefficient obtained by comparing each MS subject, each arm and task with the correspondent control subjects were considered representative of the degree of similarity between the MS group and their respective control groups. To obtain a reference value for the degree of similarity, the spinal maps of each control subject within the same arm and task were compared with the spinal maps of all other control subjects using Pearson's correlation coefficient and then averaged across the control subjects (ρ_2DINTRA−GROUP_) (Pellegrino et al., 2020, 2021a). In the same way, for each task, we estimated the similarity between arms (ρ_2D−ARM_) within groups for each population.

##### Muscle Synergies

The same preprocessed EMG dataset used for the spinal maps was used to extract the muscle synergies for each subject, task, and arm. In detail, we applied the non-negative matrix factorization (NNMF) algorithm (Lee and Seung, 2001; Tresch et al., 2006; Cheung et al., 2009) to a matrix obtained by concatenating for each muscle (rows), the normalized EMG envelopes related to the eight directions (columns) averaged over the five repetitions. The NNMF algorithm extracts from the EMG envelopes a defined number of positive components or muscle synergies, represented by a matrix of weights (W) accounting for the participation of each muscle in each synergy, and a matrix of activation coefficients (H) representing the timing of activity of each muscle synergy. For each subject, to objectively determine the minimum number of muscle synergies required to reconstruct the data set, we used the common or the higher number obtained from two different methods based on the inspection of the R^2^ curve that represents the fraction of total variation explained by the synergy model (d'Avella et al., 2006). The first method estimated the minimum number of synergies that achieved an R^2^ > 90% (Tresch et al., 2006). The second method instead is detecting a change in the slope of the R^2^ curve (Berger and d'Avella, 2014). For the second method, a series of linear regressions were performed on the portions of the curve included between the N-synergy (N = 1 to 16) and its last point (i.e., 16th synergy). N was then selected as the minimum value for which the mean squared error of the linear regression was <10^−4^. In case of a mismatch between the two criteria, the larger N was chosen (Berger and d'Avella, 2014). The same number of muscle synergies was retained across subjects within the same arm, task and group to compare the weights and activation coefficient vectors of muscle synergies among populations; the number was established as the rounded average across subjects (Coscia et al., 2014). To compare muscle synergies among tasks, arms, and groups, the weight coefficients of each muscle synergy were ordered according to their matching with a set of reference weight coefficients (Pirondini et al., 2016; Pellegrino et al., 2018) using the highest normalized scalar product between the two vectors for each task (d'Avella et al., 2003). To obtain the reference muscle synergies, for each condition and each arm separately, we created a set of reference muscle synergies by first pooling together the weight coefficients related to the right and left arms for control subjects. Then, according to Cheung et al. (2012), we used a hierarchical clustering procedure based on the minimization of the Minkowski distance between vectors to categorize them. The number of clusters was equal to the number of muscle synergies extracted for each task. We obtained the set of reference muscle synergies by averaging the synergy vectors within each cluster. Then, we ordered the synergy vectors from each subject, in each task, and in each arm separately, with respect to the set of reference muscle synergies obtained.

To assess the similarity between groups in all tasks and arms for the weight coefficients, we computed the scalar product (DOT_GROUP_) between the synergy vectors of each arm and task of the two groups, and then we calculated the mean values across subjects and synergies. In the same way, we estimated the similarity between sides (DOT_ARM_) within each group for the AF, NF, RF, and IF tasks. We evaluated the similarity of the activation coefficients of muscle synergies by using the Pearson correlation (Tropea et al., 2013). Analogously to the weight coefficients, we estimated the similarity between groups (r_GROUP_) and between arms (r_ARM_) in all tasks. To obtain a reference value to assess the degree of similarity between groups, the weight and the activation coefficients of each control subject were compared, respectively, with the weight and activation coefficients of all other controls and then averaged across muscle synergies and across individuals (DOT_INTRA−GROUP_ and r_INTRA−GROUP_, respectively) (Pellegrino et al., 2018, 2020). To evaluate the effect of task and directions on the coefficients' activation profiles (H), we calculated their root mean square value (RMSsyn) for each direction and task (Coscia et al., 2014; Pellegrino et al., 2020).

### Statistical Analysis

To test if the indicators related to behavioral performance, the spinal maps and the number and similarity measures of muscle synergies differed between the two subject groups and depended on the task or on the arm dominance, we ran repeated-measures analyses of variance (r_ANOVA_) with two within-subjects' factors: “task” (1–4: FS, AF, RF and IF), “arm” (1–2: D and ND arm); and one between groups factor, “disease” (1–2: C and MS). Furthermore, to investigate if the indicators of similarity between the two body sides in terms of behavioral indicators, spinal maps and muscle synergies (i.e., DOT_ARM_ and r_ARM_) differed between the two subject groups and depending on the task, we ran a r_ANOVA_ with one within-subjects' factors: “task” (1–4: FS, AF, RF and IF), and one between groups factor, “disease” (1–2: C and MS).

The assumption of sphericity was tested on each variable using Mauchly's test. If the assumption was rejected the Greenhouse-Geisser correction was applied. *Post hoc* analysis (Fisher's LSD test) was used to verify statistically significant differences obtained with repeated measures ANOVA. The significance level was set at *p* < 0.05. The statistical analysis was performed within the Statsoft environment.

## Results

*The two populations had similar task performance when relying on hand movements in the dynamic exercises, while relevant differences emerged when relying on the force applied by the hand in the static exercise*.

**Figure 2:**
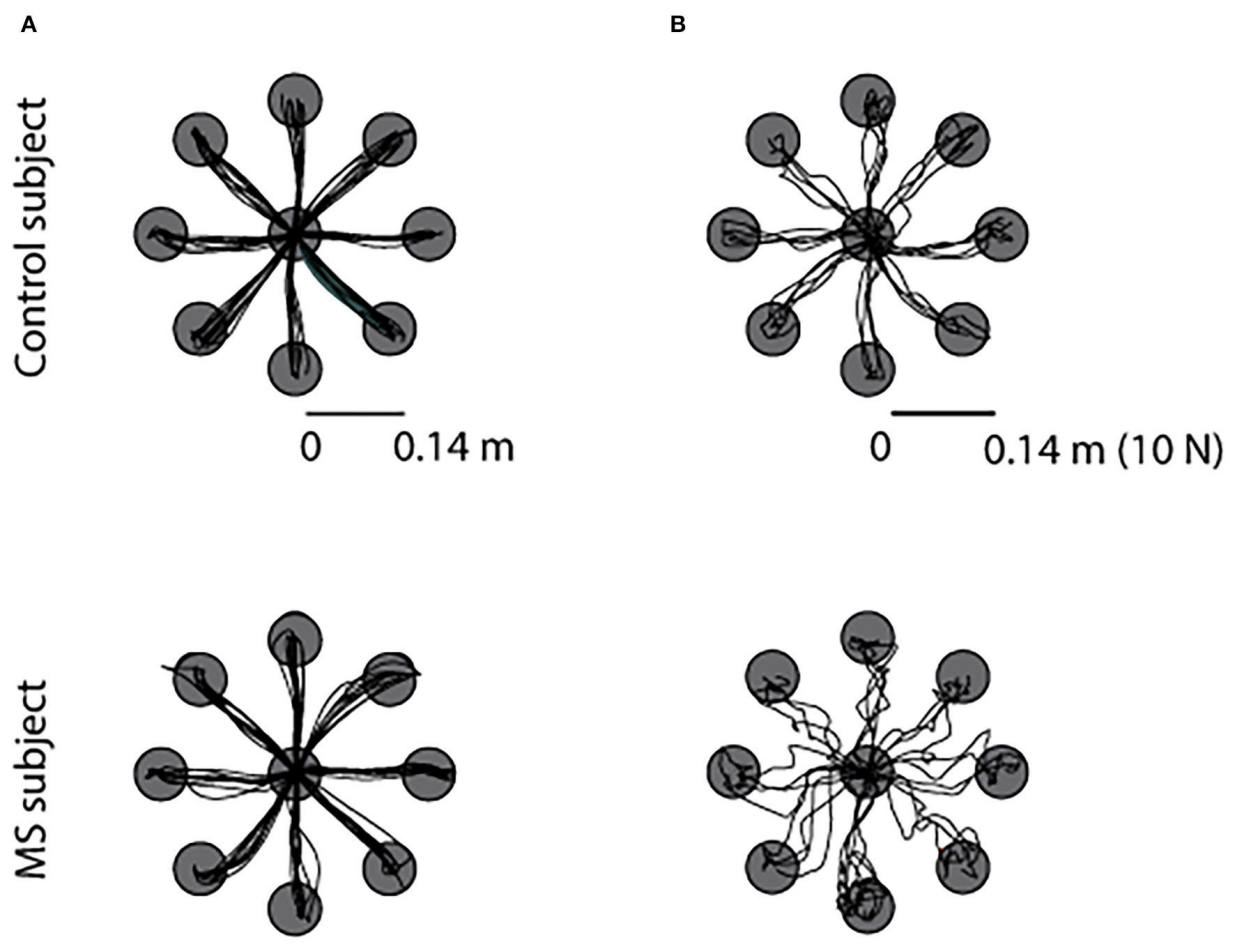
Example of trajectories of the cursor movement toward the eight targets during the null force task **(A)** and during the static force task **(B)** for a MS subject (bottom row) and the matched control subject (top row). All subjects executed the same task with the left-ND (left column) and with the right-D arm (right column), for sake of simplicity we reported the data of the tasks performed with the right-dominant arm.

As expected, from the visual inspection of cursor trajectories we could not notice relevant differences between asymptomatic MS subjects and their controls when performing a movement task. While differences emerged from the cursor trajectories generated during the force task. An example displaying the cursor trajectories of an MS and the matched control participant is shown in Figures 2A,B, for the movement in the null field and for the force exerted in the static task, respectively. In the latter case, with respect to controls, the MS subject had the cursor trajectories corresponding to the force profiles more entangled, with several overshoots of the target, especially when performing the task with the ND arm.

**Figure 3:**
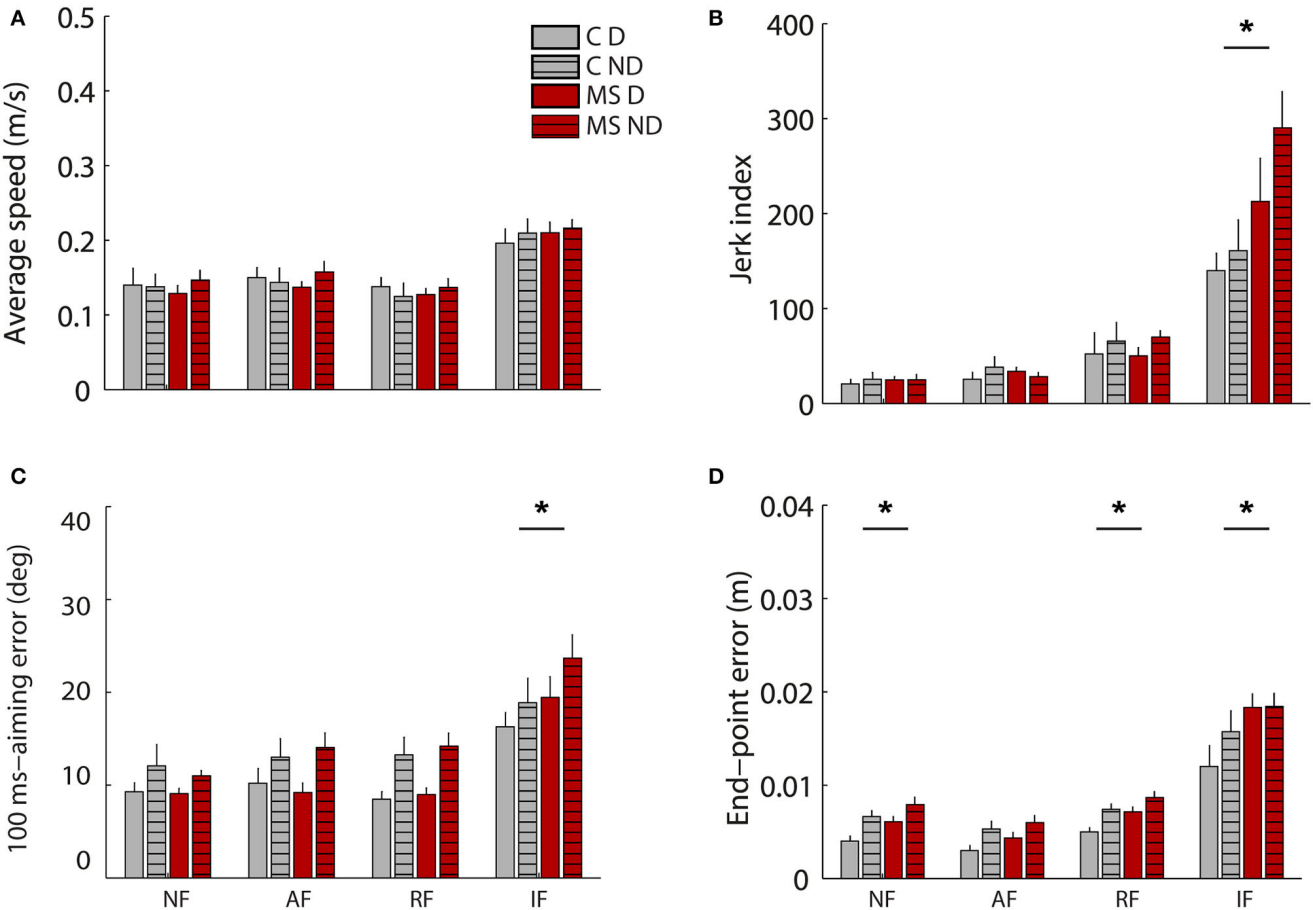
Behavioral indicators of the cursor trajectories during the motion tasks (motion trajectories) in absence of external force (NF), in presence of assistive (AF) or resistive (RF) force and during the isometric task (IF, force profiles). In particular average speed **(A)**, Jerk index **(B)**, 100ms-aiming error **(C)** and end-point error **(D)**. Control subjects **(C)** and MS subjects (MS) are shown respectively with gray and red colors as indicated in the legend. Solid colored bars and striped bars represent the right-D and left-ND arm. The error bars indicate the standard error of the indicators. *Indicates significant differences (*p* < 0.05) between subject groups (C vs. MS) for each task.

The two populations performed the required tasks at a similar speed (C: 0.156 ± 0.011 m/s SE, MS: 0.157 ± 0.013 m/s SE). No significant effect of the pathology emerged when considering all tasks for all indicators (Figure 3). Asymptomatic MS subjects and their controls had more similar performance when controlling the cursor with their hand motion, than with their force in the static task. Indeed, in the latter case, MS participants with respect to their controls generated less smooth [disease x task: jerk index F(3,36) = 0.32 *p* = 0.021] and accurate trajectories both in the initial (planning) part of the trajectory and at the end of the first sub-movement [100-ms aiming error F(3,36) = 2.59 *p* = 0.03, end-point error F(3,36) = 1.49 *p* < 0.001] particularly when controlling a force trajectory, IF task (*post hoc* jerk: *p* = 0.036, 100-ms aiming error *p* = 0.026, end-point error *p* = 0.006). The difference between MS and control subjects in the end-point error resulted statistically significant also for the NF (*p* = 0.026) and the RF tasks (*p* = 0.014).

Additionally, a slight, but significant asymmetry between the two sides of the body was observed for all participants, both MS and controls: there was a significant effect of the arm for the 100-ms aiming error [F(1,12) = 33.93 *p* < 0.001]. No significant differences between the two populations related to the similarity of performance between the two sides of the body were observed.

*Asymptomatic MS participants had different muscle activation patterns than controls when exerting forces or when moving in presence of external forces generated by a robot. These differences were not evident when they moved their arm in absence of external forces*.

**Figure 4:**
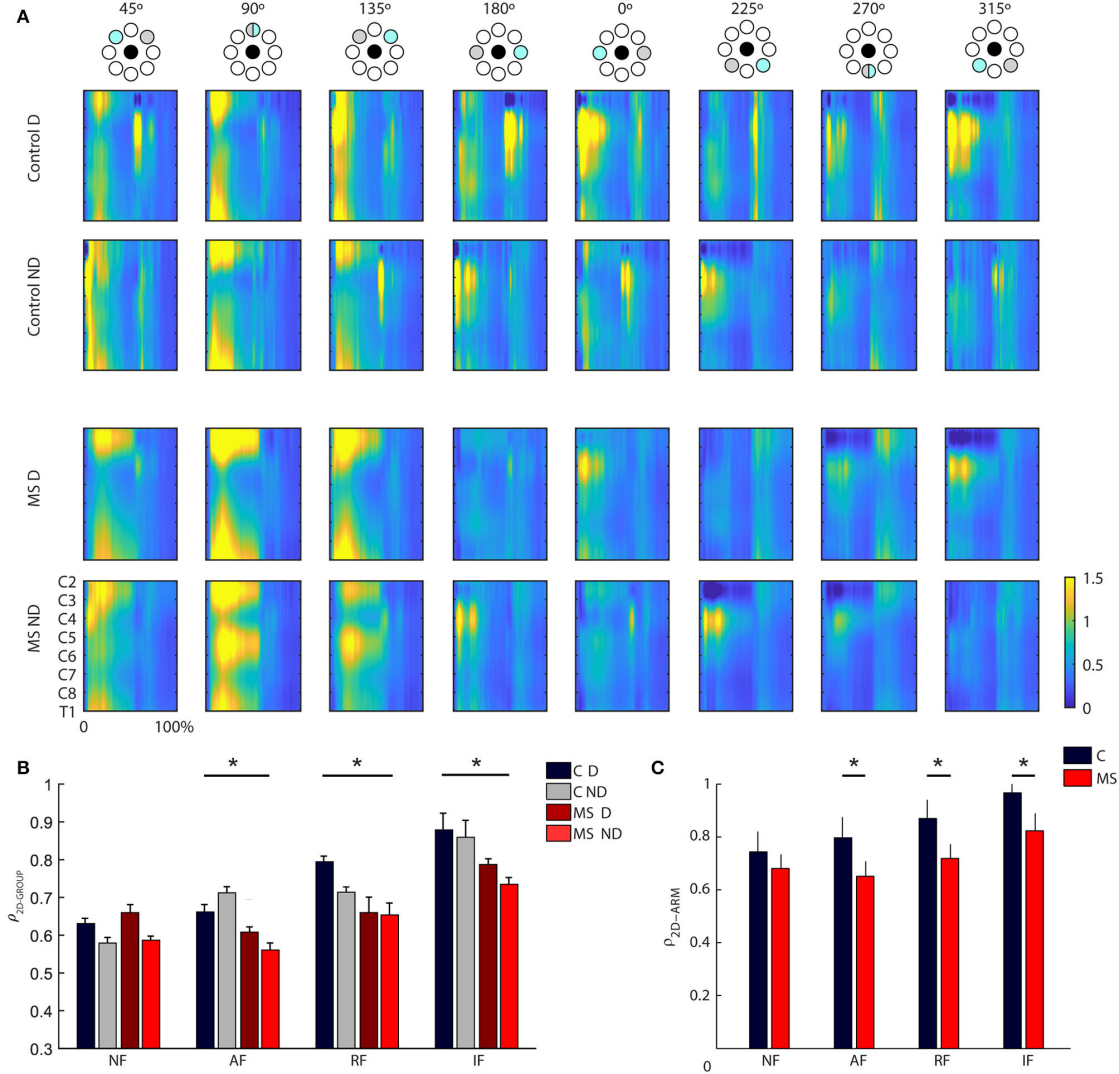
**(A)** Spinal maps obtained from the resistive field task for control (first two rows) and MS subjects (last two rows) divided by directions and arm (left-ND and right-D). On the x-axis, the trial duration is represented as a percentage. Spinal maps refer to equal movements in the joint space, i.e., for each column, the top panel indicates the corresponding target directions (gray target) for the right arm, while the corresponding target directions of the left arm were mirror symmetric with respect to the vertical midline. **(B)** Mean and standard error of the inter-group similarity (ρ_2D−GROUP_) between control and MS subjects in presence of assistive (AF), resistive (RF), isometric (IF) and in absence of external force (NF) for the dominant (solid-colored bars) and non- dominant (striped bars) arm. The gray bars, solid colored and striped, reflect the intra-group degree of similarity in the control group respectively for the D and ND arm. **(C)** Mean and standard error of the between arm similarity (ρ_2D−ARM_) for controls (C, gray bar), and multiple sclerosis subjects (MS, red bar) in presence of assistive (AF), resistive (RF) and in absence of external force (NF). ^*^Indicates significant differences (*p* < 0.05).

As for the analysis of muscle patterns, first we used spinal maps as a tool to describe the spatiotemporal organization of the muscle signals at the level of the spinal cord. In control subjects, the spinal maps were characterized by a main activation from 10 to 60% (Figure 4A) of the trial, and from 10 to 100% in the static task (see Supplementary Material). This activity was observed between C3 and C6 for the lateral (right-left, i.e., 0° and 180°) directions and for the directions closer to the body of the subjects (i.e., 270° and 315°) while the activity was more diffuse along all the spinal segments in the directions toward targets further away from the subject (i.e., 45° 90° 135°); see Figure 4A. The MS subjects had a different organization of the spinal maps with respect to control subjects confirmed by differences in the 2D Pearson's correlation coefficient ρ_2D−GROUP_ [F(1, 12) = 8.31 *p* < 0.001; disease x task effect: F(3, 36) = 19.5, *p* < 0.001; see Figure 4B], mainly due to the prolonged main burst of the spinal map activity for MS subjects, especially in the directions involving an extension of the arm (i.e., 45°, 90°, 135°) for the RF, NF, and AF tasks (all *post hoc* tests *p* < 0.001), see Supplementary Material for spinal maps of NF, AF, and IF tasks. This was observable in both the dominant and non-dominant arm. Additionally, for both populations, there was a significant effect of the arm [F(1,12) = 7,0 *p* = 0.019] that consisted in a more diffuse activity along all the cervical levels while moving the dominant arm for the directions involving arm extension toward targets on the opposite side of the arm used to execute the task (i.e., 90° 135° 180°). The spinal maps of the two arms were more similar for the controls than for the MS subjects [ρ_2D−ARM−_pathology effect: F(1,12) = 18.47, *p* = 0.01, see Figure 4C]. Finally, the mechanical environment determined significant differences in the spinal maps when comparing arms of the same subject [ρ_2D−ARM−_ disease x task effect: F(3, 36) = 10.9, *p* = 0.001; *post hoc* AF and NF: *p* = 0.01 and IF: *p* = 0.02]; see Figure 4C.

*Asymptomatic MS and control subjects, had same number and similar structure, but different activation profiles of the muscle synergies*.

The number of synergies was not significantly different between populations [F(1,12) = 3.7, *p* = 0.46] and between the dominant and non-dominant arms for both populations [F(1,20) = 1.77, *p* = 0.38]. The number of synergies was different among tasks [task effect: F(3,36) = 98.69 *p* = 0.001]. In the dynamic tasks: NF, AF, and RF tasks five muscle synergies were extracted for dominant (NF: 5.14 ± 0.4SE, AF:4.94 ± 0.34SE and RF: 5.07 ± 0.42SE) and non- dominant (NF: 4.97 ± 0.29SE, AF: 4.65 ± 0.34SE and RF: 5.21 ± 0.42SE) arm of control subjects and for D (NF: 5.42 ± 0.20SE, AF: 5.47 ± 0.42SE and RF: 5.41 ± 0.36SE) and ND arm (NF: 4.68 ± 0.47SE, AF: 5.25 ± 0.45SE and RF: 5.11 ± 0.74SE) of MS subjects. While in the IF task, only four muscle synergies were identified for dominant (control 3.52 ± 0.48 MS subject 3.85 ± 0.26) and non-dominant (control 4.01 ± 0.30 MS subject 3.52 ± 0.36) arm of both populations.

**Figure 5:**
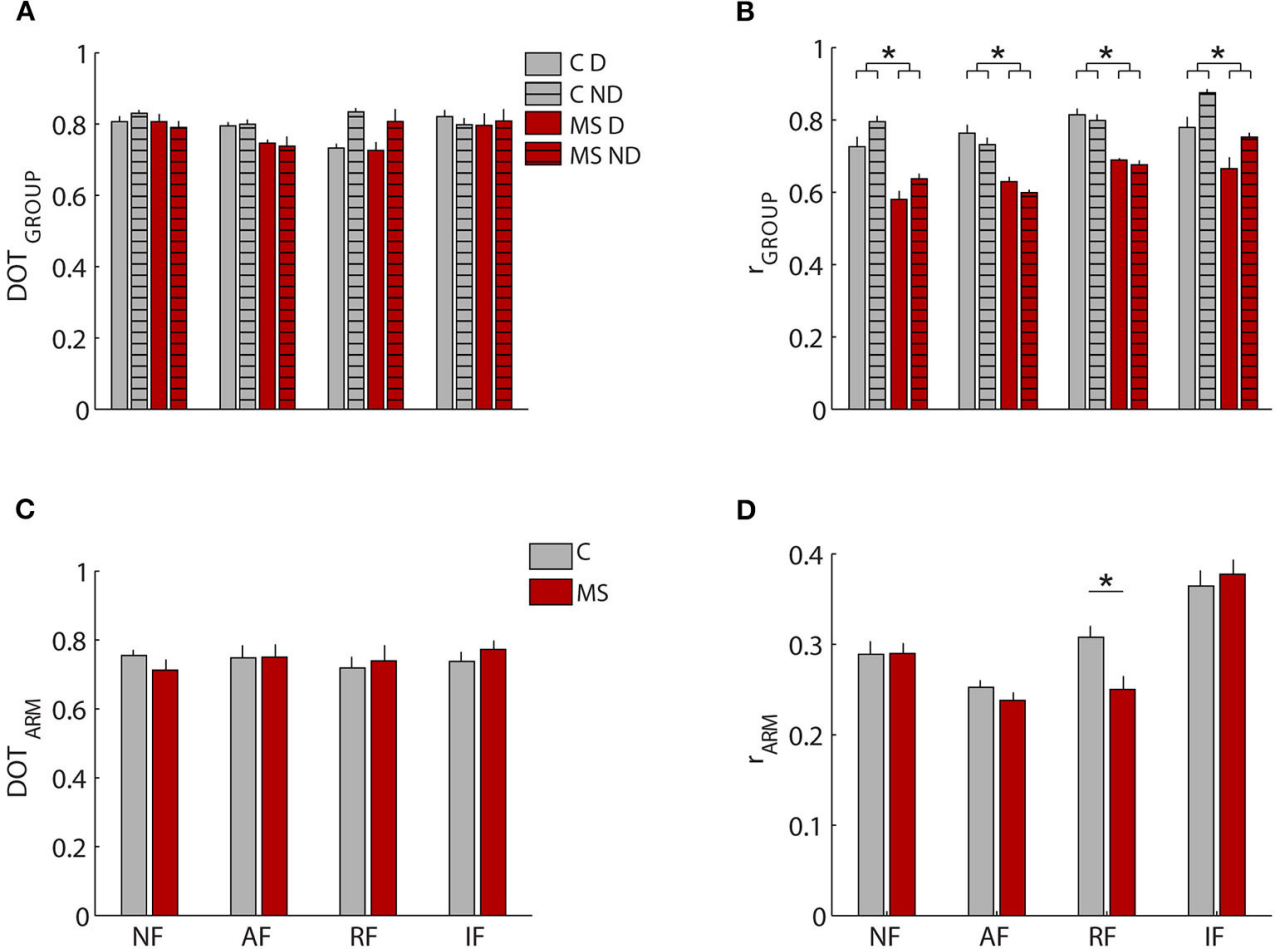
Comparison of weight coefficients of muscle synergies by the scalar product (DOT) and activation coefficients by Pearson correlation (r). **(A,B)** MS subjects (red bars) compared to control subjects (gray bars), i.e., inter-groups indicator for the dominant (D, solid-colored bars) and the non-dominant (ND, striped bars) arm compared to the same indicator computed intra-group. **(C,D)** comparison between the two arms for MS subjects (MS, red bars) and control subjects (C, black bars). The error bars represent the standard errors. ^*^Indicates significant differences (*p* < 0.05) between subject groups.

The organization of the muscle synergies in response to the mechanical environment that characterized each task did not change among subjects of the two populations and between arms. Indeed, we did not find a significant global difference when comparing the DOT_INTER−GROUPS_ obtained from the comparison between MS and control subjects and the DOT_INTRA−GROUP_ obtained from the comparison within the control group [F(1,12) = 0.97, *p* = 0.41; Figure 5A]. In the comparison of D and ND arm (DOT_ARM_), we did not find any difference in weight coefficients of the dominant and non- dominant arm (DOT_ARM_) of both controls and MS subjects in all tasks [F(1,12) = 0.74, *p* = 0.34; Figure 5C].

Conversely, when investigating the synergies temporal activations comparing by Pearson correlation, the activation profiles of muscle synergies between the two populations (r_GROUP_ Figure 5B) and between D and ND arm (r_ARM_, Figure 5D), we detected relevant differences. As for the comparison between the two populations, we found a significant difference in the activation profiles of muscle synergies between the r_INTER−GROUPS_ and r_INTRA−GROUP_ [F(1,12) = 4.891, *p* < 0.047; Figure 5B], The synergies 2-3-4 had the greater differences between groups as described below.

**Figure 6:**
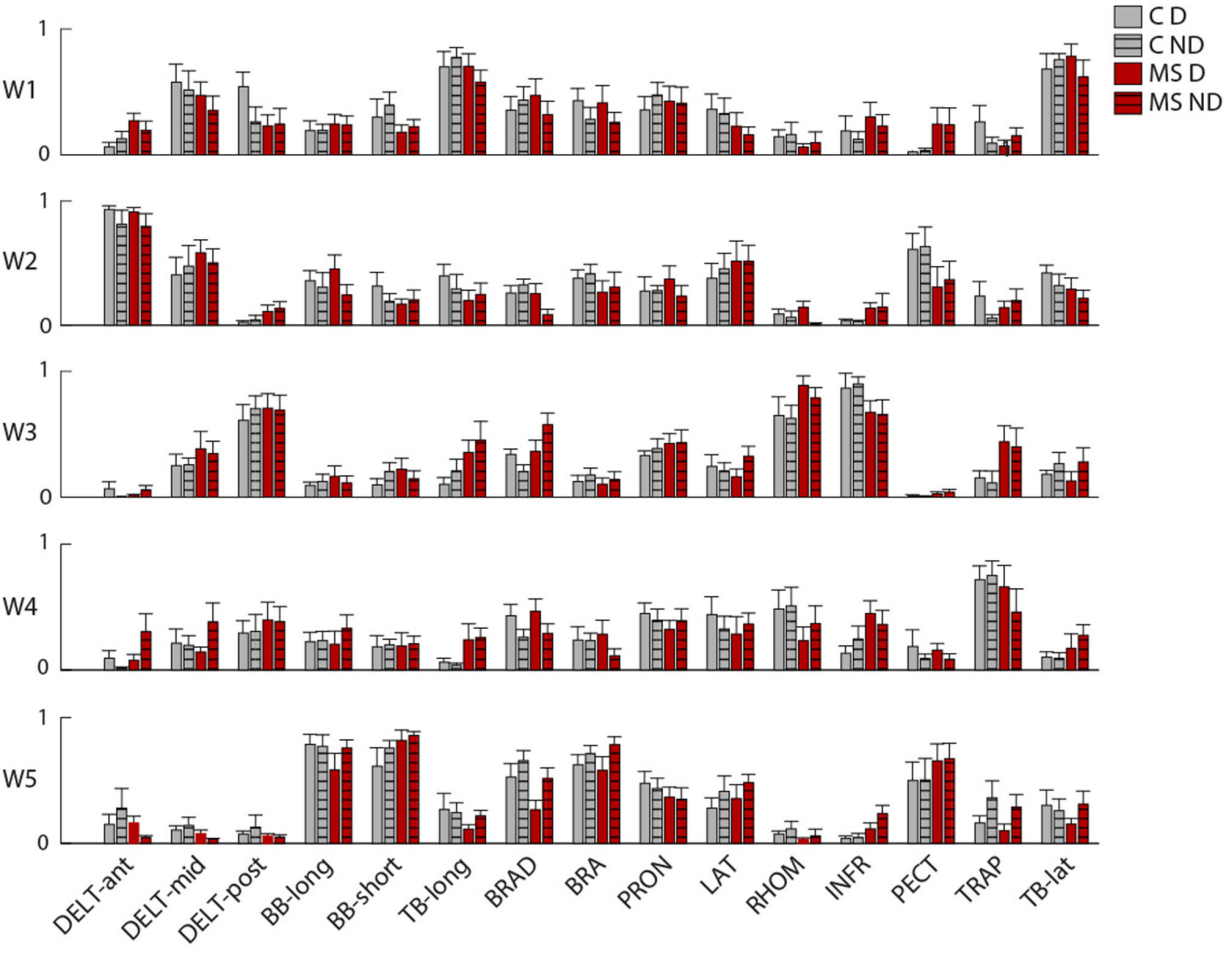
Weight coefficients of the muscle synergies during the task in absence of external forces (NF). Weight coefficients (W1 to W5) are shown for the two arms (solid colored: right-D and striped: left-ND). Control subjects (C) and MS subjects (MS) are shown with different colors as indicated in the legend, gray and red. The error bars represent the standard error.

**Figure 7:**
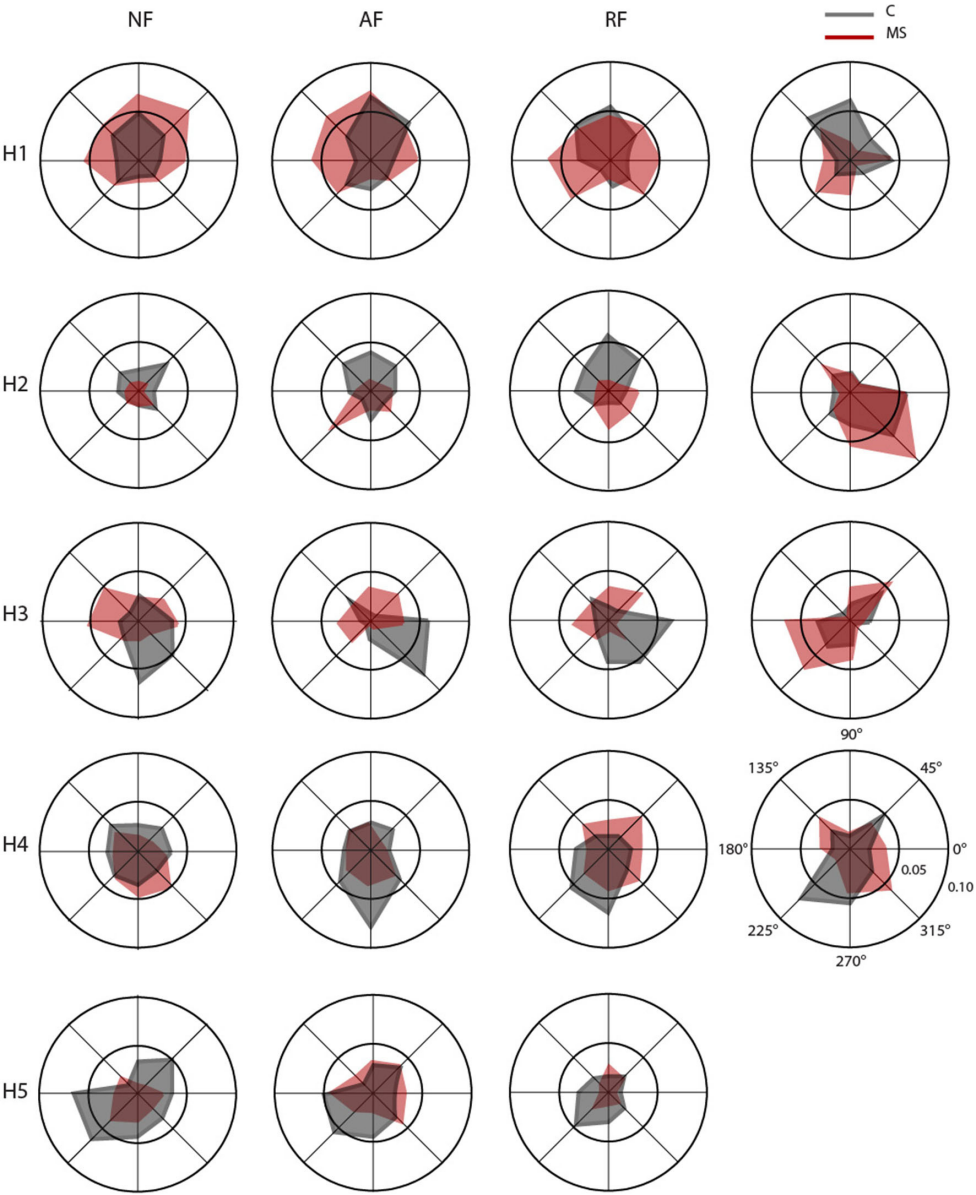
Polar plots of mean RMS for the activation profiles coefficients of the muscle synergies (H1–H5) in null force (NF, first column), assistive force (AF, second column), resistive force (RF, third column) and isometric force (IF, fourth column). Each radial line represents one of the eight directions. For each direction, mean RMS of the activation profile coefficients for control (C, gray) and multiple sclerosis (MS, red) subjects, respectively.

In Figure 6, we reported an example of weight coefficient and in Figure 7 the RMS of the activation profiles of the 5 extracted muscle synergies for the NF task. In particular, the following synergies were extracted:

Synergy 1 principally involved the activity of muscles controlling the upper arm during elbow extension for both arms (TB-long and TB-short). It was mainly active during movements/force exertion directed toward 45°, 90°, and 135°. For asymptomatic MS subjects, in the dynamic tasks, it was also active during movements toward 0° and 180°.

Synergy 2 included the activity of muscles controlling the upper arm during horizontal shoulder abduction and extension, it principally involved the DELT-ant and DELT-med, with contributions from other muscles (i.e., LAT and PECT). For controls, this synergy in the dynamic tasks was mainly active toward 90° and 45° while in the isometric task was mainly active for forces exerted toward 0° and 315°, while in asymptomatic MS participants for the RF and IF tasks, it was mainly active in the directions 270° and 315°.

Synergy 3 principally involved the activity of muscles controlling the upper arm during horizontal shoulder adduction and flexion (DELT-post, RHOM, and INFR). For controls, it was mainly active during movements/force exertion directed toward 0°, 270°, and 315°, while for the asymptomatic MS participants in the dynamic tasks, it was mainly active in the directions 45°, 180°, and 135°.

Synergy 4 principally involved the TRAP, with minor contributions from other muscles. In control subjects, it facilitated the stabilization of the shoulder in the movement/force toward the body, i.e., 225°, 270°, and 315°, while in asymptomatic MS, the activation of this synergy was more similar in the forward and backward directions.

Synergy 5 principally involved muscle participating in the flexion of the arm and adduction of the shoulder (BB-long, BB-short and PECT). Synergy 5 was more active during movements directed toward the body of the subject, mainly in the 225°- 270° directions. This directionality of the activation profiles was not observed for asymptomatic MS subjects.

In the IF task, synergy 5 was absent and the contribution of the BB-long and BB-short was distributed in the other synergies; while the activity of PECT muscle was presented in synergy 2.

As for comparison between the D and ND sides, the correlation between the activation profiles of the two arms (r_ARM_) for both groups did not result in a significantly different [F(1,12) = 0.263 *p* = 0.617] Figure 5D. The limited number of subjects did not allow significant differences between arms seen in previous works to emerge, but it was sufficient to highlight differences between MS and control subjects.

## Discussion

Besides fatigue, motion, and cognitive deficits, upper limb dysfunction is one of the important characteristics of people with multiple sclerosis (MS). In the early phase of the disease, MS subjects might be asymptomatic, the disease might be silent, and therefore, subjects do not present any appreciable deficit. In this study, we wanted to test if a protocol, using a robotic manipulandum and an EMG system, and a set of indicators taking into consideration not only behavioral but also muscular and motor coordination parameters allowed the identification of differences in motor abilities between asymptomatic MS subjects and their matched control subjects. We found that subtle differences are detectable using such in-depth analysis. Task performance based on the movement ability or force control suggested that a force task is more sensitive to differences due to the disease in MS people with no evident disability. The cursor trajectories generated during this force task were different between the two populations on visual inspection (Figure 2B), while the trajectories generated during the dynamic tasks (NF, AF and RF) were more similar (Figure 2A). In fact, force trajectories generated by asymptomatic MS subjects were less smooth and accurate than those of their controls, as already highlighted in Vergaro et al. (2010) and Pellegrino et al. (2018). Moreover, in accordance with previous works (Casadio et al., 2007; Solaro et al., 2007; Vergaro et al., 2010; Valè et al., 2020), MS subjects executed more inaccurate movements with end-point errors higher that controls in a both force and movement tasks.

The fact the IF task allowed to detect difference not observable in movements tasks might be due to the fact that force and motion are likely to have separate neural representations (Casadio et al., 2015). Thus, it could be more challenging to modulate a force exerted by the hand rather than moving the arm in the workspace, i.e., the ability to control forces in isometric conditions could be earlier and /or more affected by the disease onset, revealing abnormalities in the related motor commands. Therefore, this task could be further explored as sensitive tool to detect differences between stages of the disease also in the early phase.

As for the muscle patterns, we found that MS might influence the ability to generalize and adapt muscle activation patterns in different movement and force tasks even when the disease did not manifest its evident motor symptoms. In fact, the spinal maps highlighted additional differences between MS and control subjects. Few studies applied this method to upper limb movements of subjects with neuromotor disabilities (Coscia et al., 2015; Pirondini et al., 2016; Pierella et al., 2020; Pellegrino et al., 2021a,b), and to the best of our knowledge, none was focused on asymptomatic MS subjects. We found that spinal maps were sensitive to MS also in case of asymptomatic subjects. They confirmed that the IF task represents a suitable task to detect muscle activity deficits between MS and control subjects, with different muscle patterns than in the RF task.

These differences in modulation and activation of specific muscle groups in MS subjects suggested to further investigate the coordinated activity of groups of muscles, i.e., the muscle synergies.

We did not find any difference in the number of synergies between asymptomatic MS and control subjects. This was expected because it is in accordance with our previous study with symptomatic MS subjects (Pellegrino et al., 2018) and with the literature (Clark et al., 2010; Cheung et al., 2012) suggesting a correlation between the dimensionality of muscle synergies and the level of the neurological motor and functional impairments. The latter observation steamed from studies in stroke survivors, e.g., Cheung et al. (2012) found that mildly impaired stroke survivors have their muscle synergies similar to the control subjects. Moreover, the number of muscle synergies both in the movement and isometric tasks was consistent with what has been already found by others with similar protocols (d'Avella et al., 2006; Roh et al., 2013; Pellegrino et al., 2018). The isometric task was characterized by a smaller number of synergies than the movement tasks for both populations.

Also, the MS and control subjects did not show significant differences in the structure of their muscle synergies. For both populations, the analysis identified two primary synergies which involved the distal muscles, one synergy that involved proximal muscles and two synergies included shoulder muscles, as also observed by other authors (Flanders and Herrmann, 1992; d'Avella et al., 2006).

Conversely, the temporal activations of the muscle synergy for asymptomatic MS subjects, as for symptomatic MS subjects (Pellegrino et al., 2018), differed from that of the controls in all tasks. Indeed, the activation of the muscle synergies was less direction-specific than in controls. These results are in line with the literature that used muscle synergy analysis to highlight muscle coordination deficits in symptomatic MS (Pellegrino et al., 2018) or other neurological conditions like stroke (Safavynia, 2011; Cheung et al., 2012). In fact, as in our case, specific synergies dominated by the activation of shoulder muscles were altered both in chronic (Roh et al., 2013) and acute (Tropea et al., 2013; Pierella et al., 2020) stroke subjects with moderate impairment of the upper limbs.

In conclusion, taken together, these findings suggest that the combined analysis of behavioral and muscle activation patterns could improve the understanding of motor impairment in subjects with asymptomatic MS and that such approach can help extracting biomarkers useful to discriminate subjects with and without MS since the early onset of the disease and in the asymptomatic phase. We demonstrated that the interaction with external forces is a powerful task to highlight meaningful functional impairments in asymptomatic MS subjects. Indeed, evident differences were visible in particular in the isometric task or when the robot applied resistive forces. The behavioral parameters describing movement quality, i.e., smoothness and accuracy, were sensitive to alterations in movement execution due to MS disease. Spinal maps and muscle synergy analysis revealed modifications of muscle activity in the MS population providing additional insights on the possible different characteristics of MS subjects with no clinical disability. A cautionary note: due to the small sample size of the population involved, this study is to consider a proof-of-concept study. Future developments will consist in enlarging the MS population with similar clinical conditions to further validate the results and have widespread indications for monitoring the onset of the first signs of sensory-motor disability and the design of proper rehabilitation protocols.

## Data Availability Statement

The raw data supporting the conclusions of this article will be made available by the authors, without undue reservation.

## Ethics Statement

The studies involving human participants were reviewed and approved by Comitato Etico Regionale Liguria, 06-10-2014, 201REG2014. The patients/participants provided their written informed consent to participate in this study.

## Author Contributions

Conceptualization, software, and formal analysis: LP, MCo, and MCa. Methodology, validation, writing—original draft preparation, and visualization: LP, CP, MCo, and MCa. Investigation: LP, MM, CS, and MM. Resources: CS, MM, and MCa. Data curation: LP and MCa. Writing—review and editing: CP, LP, MCo, MCa, MM, MI, and CS. Supervision: MM, CS, MCo, and MCa. Project administration: MM, CS, and MCa. Funding acquisition: MCa, CP, and MI. All authors have read and agreed to the published version of the manuscript.

## Funding

Research supported by the Italian Multiple Sclerosis Foundation (FISM, 2013-Cod. 2013/R/5), Marie Curie Integration Grant (REMAKE, FP7-PEOPLE-2012-CIG-334201), and European Union's Horizon 2020 Research and Innovation Program under the Marie Sklodowska-Curie Grant Agreement No 896892 (REMAp).

## Conflict of Interest

MCo was employed by confinis ag. The remaining authors declare that the research was conducted in the absence of any commercial or financial relationships that could be construed as a potential conflict of interest.

## Publisher's Note

All claims expressed in this article are solely those of the authors and do not necessarily represent those of their affiliated organizations, or those of the publisher, the editors and the reviewers. Any product that may be evaluated in this article, or claim that may be made by its manufacturer, is not guaranteed or endorsed by the publisher.
