# Supplementary material for: Upper Limb Sensory-Motor Control During Exposure to Different Mechanical Environments in Multiple Sclerosis Subjects With No Clinical Disability
Source: Front Neurorobot. 2022 Jul 11;16:920118. doi: 10.3389/fnbot.2022.920118 (PMC9309790; doi:10.3389/fnbot.2022.920118)

Supplementary Material

1 Supplementary Figures and Tables

1.1 Supplementary Figures

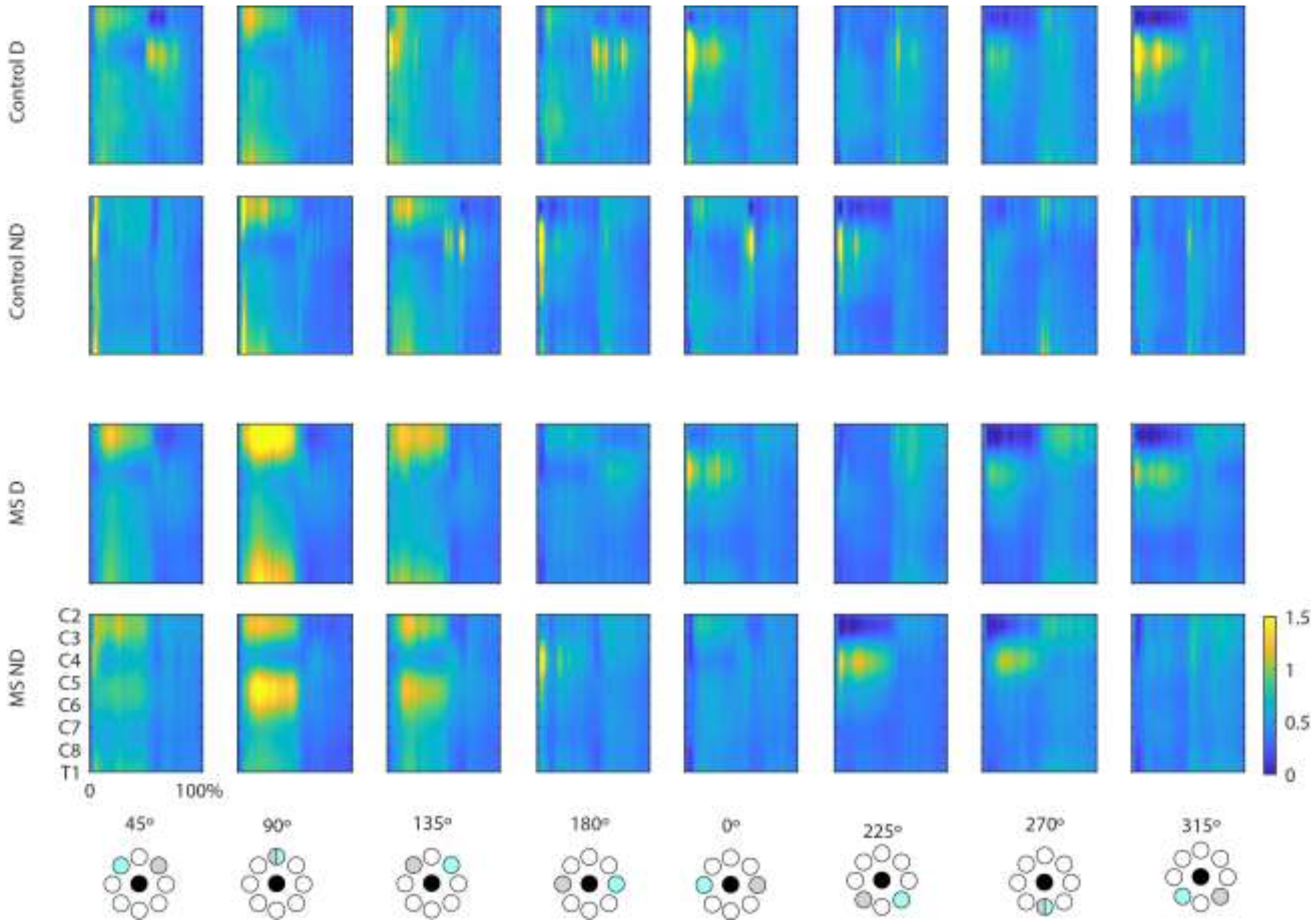

Supplementary Figure 1. The spinal maps obtained for the eight targets in the null force task (NF). The first two rows are referred to the dominant (D, right) and non dominant (ND, left) arm of control subjects, the third and the forth rows, respectively, to the D and ND arms of subjects with multiple sclerosis (MS). On the x-axis the movement duration is represented in percentage. Spinal maps are referred to equal movements in the joint space, i.e. for each column the top panel indicates the

corresponding target directions (grey target) for the right arm, while the corresponding target directions of the left arm were mirror symmetric with respect to the vertical midline.

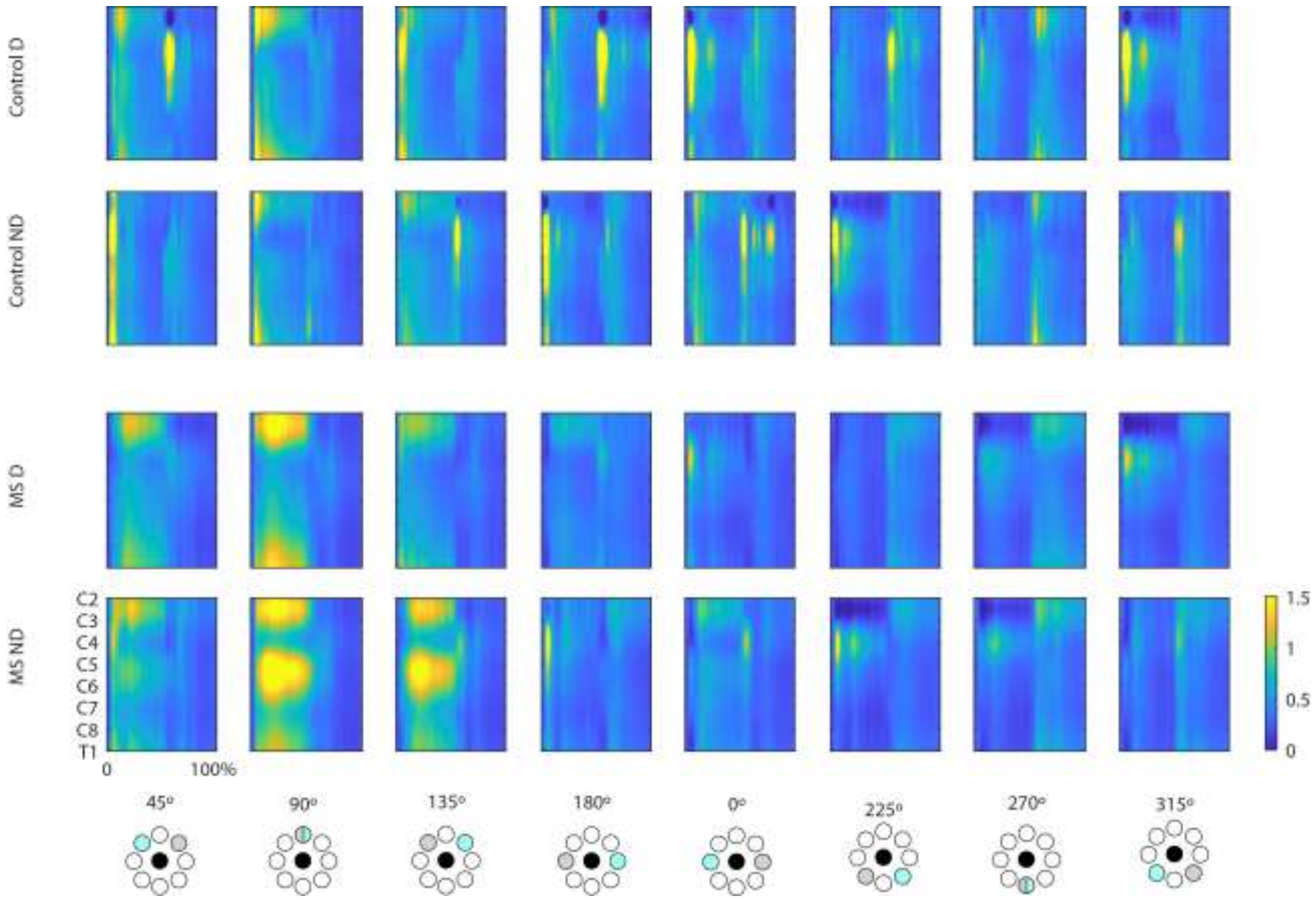

Supplementary Figure 2. The spinal maps obtained for the eight targets in the assistive force task (AF). The first two rows are referred to the dominant (D, right) and non dominant (ND, left) arm of control subjects, the third and the forth rows, respectively, to the D and ND arms of subjects with multiple sclerosis (MS). On the x-axis the movement duration is represented in percentage. Spinal maps are referred to equal movements in the joint space, i.e. for each column the top panel indicates the corresponding target directions (grey target) for the right arm, while the corresponding target directions of the left arm were mirror symmetric with respect to the vertical midline.

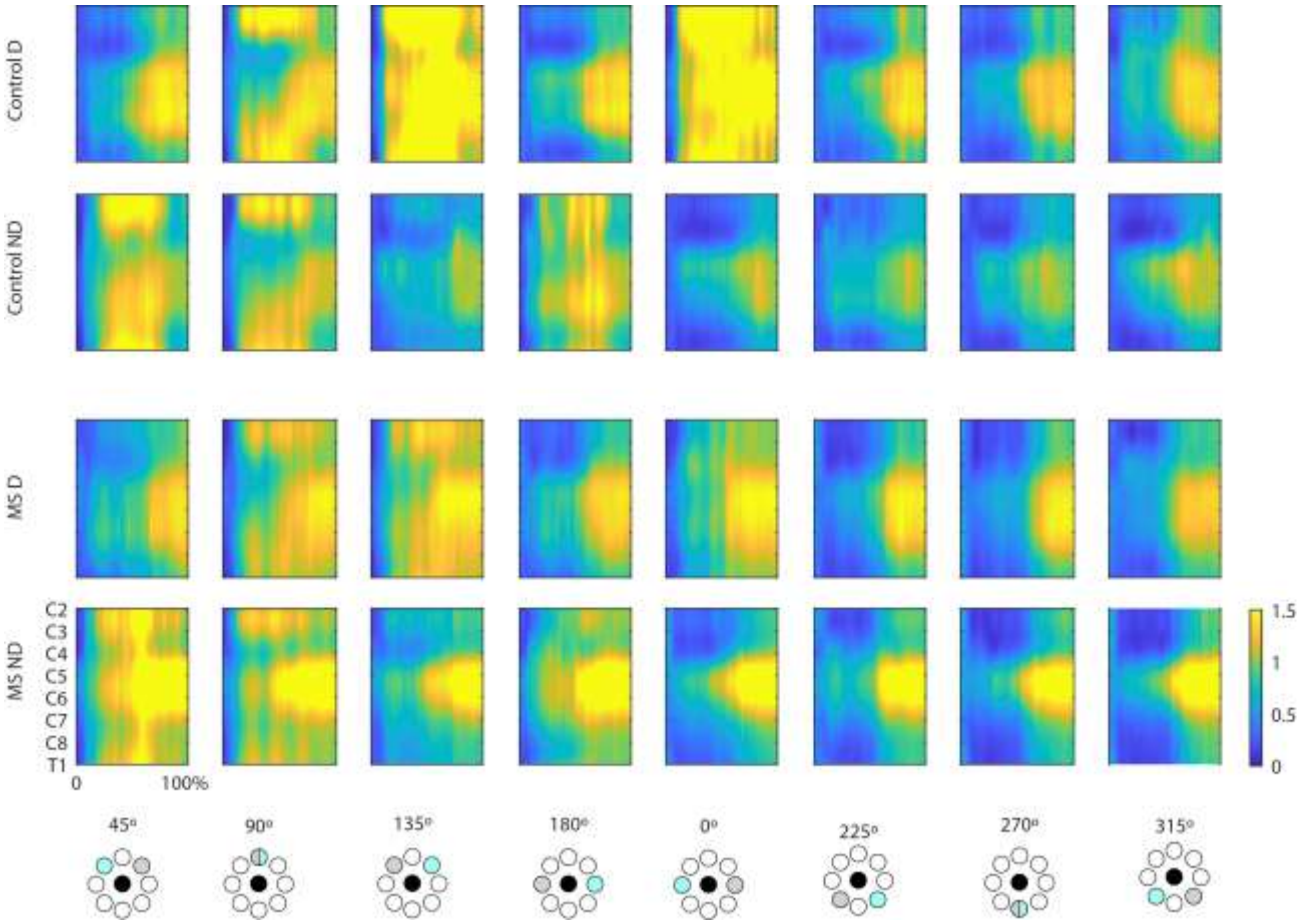

Supplementary Figure 3. The spinal maps obtained for the eight targets in the isometric force task (IF). The first two rows are referred to the dominant (D, right) and non dominant (ND, left) arm of control subjects, the third and the forth rows, respectively, to the D and ND arms of subjects with multiple sclerosis (MS). On the x-axis the movement duration is represented in percentage. Spinal maps are referred to equal movements in the joint space, i.e. for each column the top panel indicates the corresponding target directions (grey target) for the right arm, while the corresponding target directions of the left arm were mirror symmetric with respect to the vertical midline.

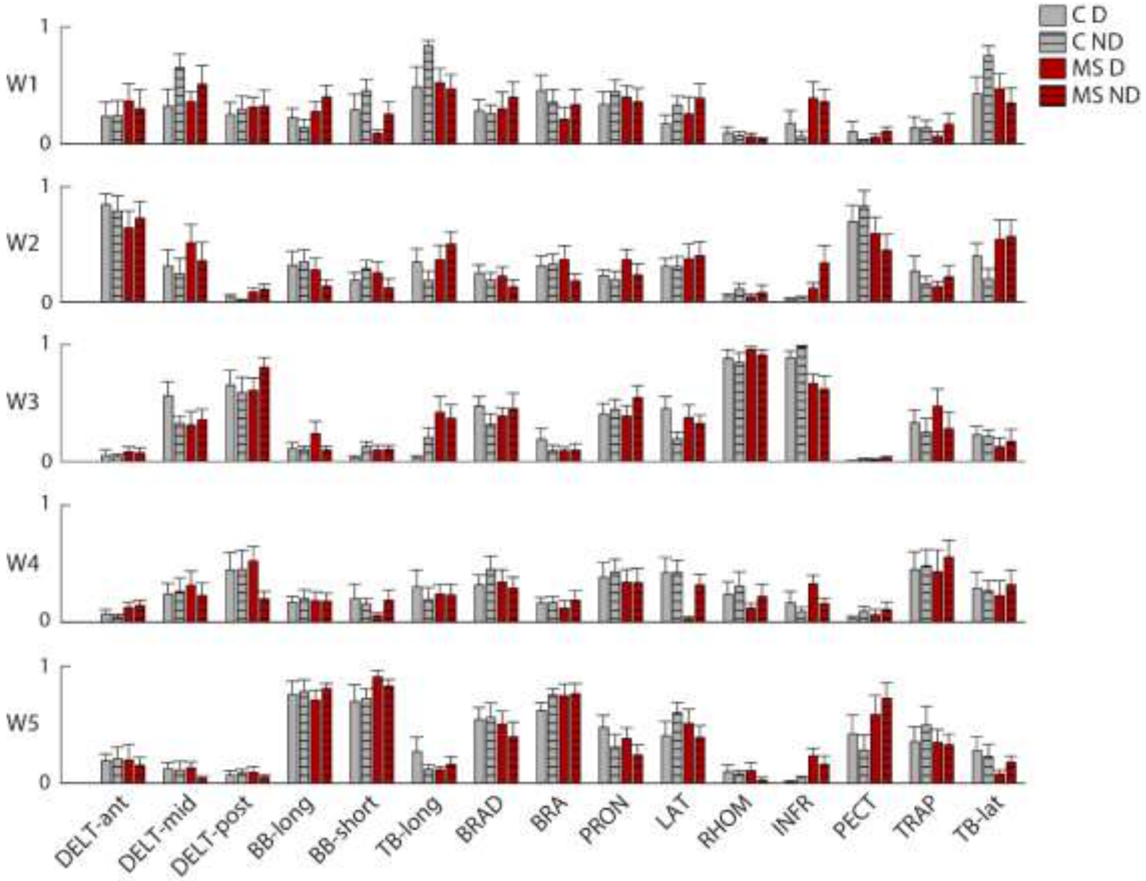

Figure 4. Weight coefficients of muscle synergies during the resistive force task (RF). Weight coefficients for all muscle synergies (W1 to W5) for the two arms (dominant D, right arm: bars with solid color; non dominant ND, left arm: striped bars). Controls (C) and MS subjects are shown with different colors as indicated in the legend. The error bars represent the standard error.

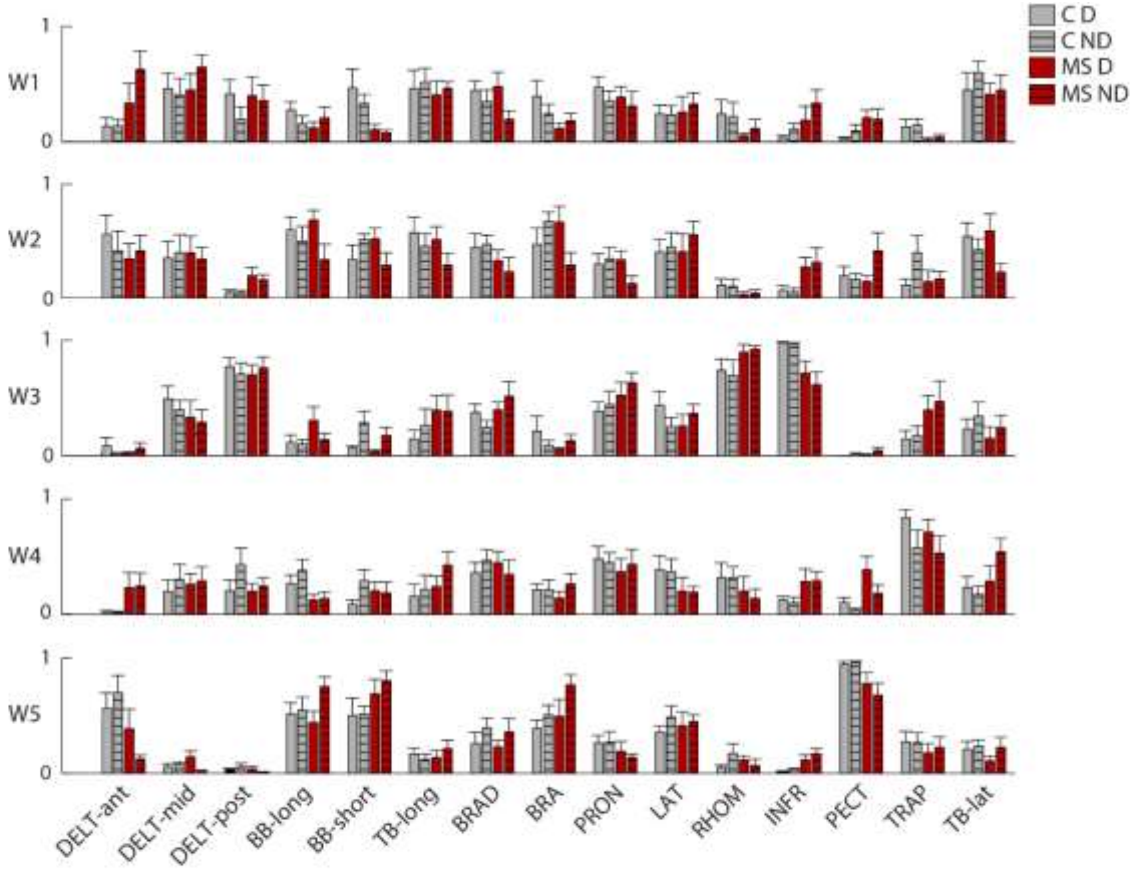

Supplementary Figure 5. Weight coefficients of muscle synergies during the assistive force task (AF). Weight coefficients for all muscle synergies (W1 to W5) for the two arms (dominant D, right arm: bars with solid color; non dominant ND, left arm: striped bars). Controls (C) and MS subjects are shown with different colors as indicated in the legend. The error bars represent the standard error.

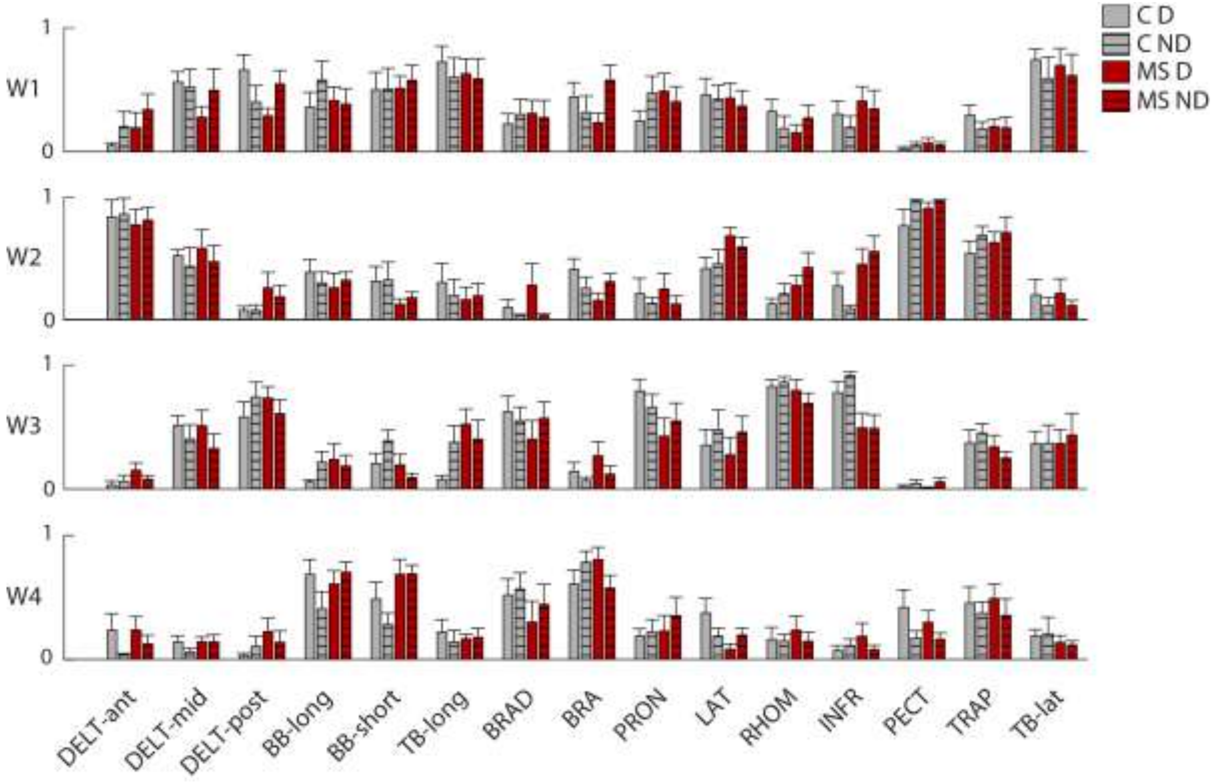

Supplementary Figure 6. Weight and activation coefficients of muscle synergies during the isometric force task (IF). Weight coefficients for all muscle synergies (W1 to W4) for the two arms (dominant D, right arm: bars with solid color; non dominant ND, left arm: striped bars). Controls (C) and MS subjects are shown with different colors as indicated in the legend. The error bars represent the standard error.

1.2 Supplementary Tables

Supplementary Table 1. Kendall's chart for the computation of spinal maps

	DELT-ant	PECT	LAT	INFR	RHOM	BB-short	BB-long	PRON	BRAD	TB-lat	TB-long	DELT-mid	DELT-post	TRAP	BRA
**C2**														x	
**C3**														X	
**C4**				x	x									X	
**C5**	X	X		X	X	X	X		X			X	X		x
**C6**	X	X	X	X		X	X	X	X	x	x	X	X		X
**C7**		X	X					X		X	X				X
**C8**		X	X							X	X				x
**T1**		X								x	x				

Supplementary Table 2. Innervation of the upper limb muscles

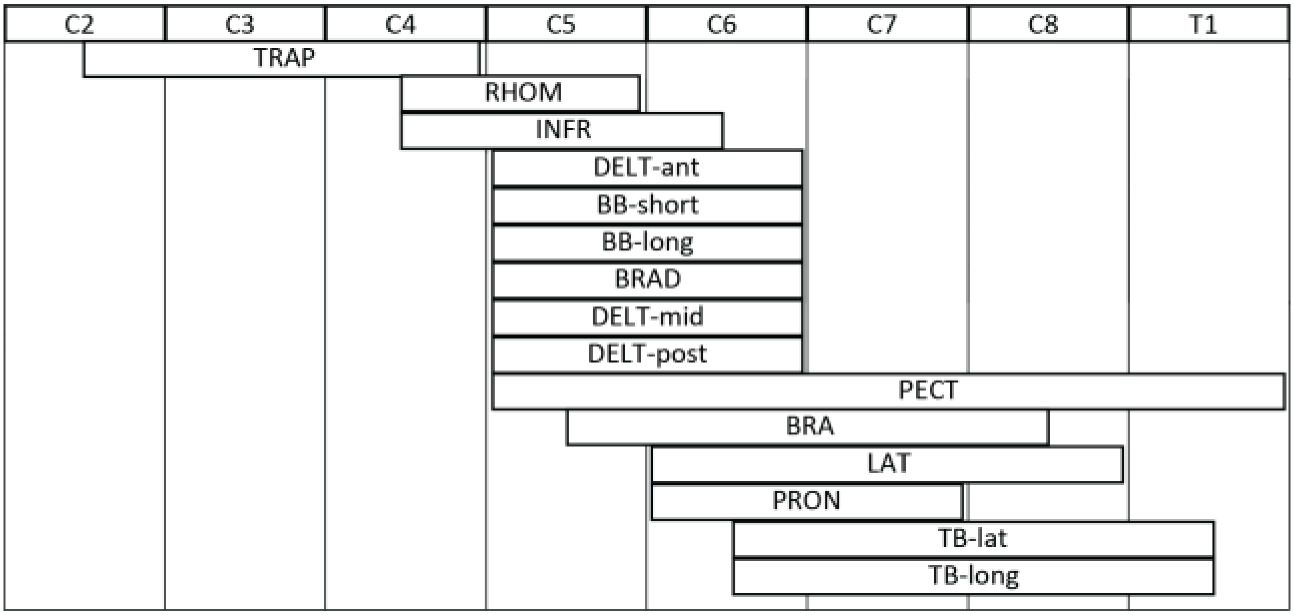

Supplement: Supplementary file 1 [file Data_Sheet_1.pdf]
